# Identification of Novel Genetic Risk Loci in Maltese Dogs with Necrotizing Meningoencephalitis and Evidence of a Shared Genetic Risk across Toy Dog Breeds

**DOI:** 10.1371/journal.pone.0112755

**Published:** 2014-11-13

**Authors:** Isabelle Schrauwen, Renee M. Barber, Scott J. Schatzberg, Ashley L. Siniard, Jason J. Corneveaux, Brian F. Porter, Karen M. Vernau, Rebekah I. Keesler, Kaspar Matiasek, Thomas Flegel, Andrew D. Miller, Teresa Southard, Christopher L. Mariani, Gayle C. Johnson, Matthew J. Huentelman

**Affiliations:** 1 Neurogenomics Division, Translational Genomics Research Institute, Phoenix, Arizona, United States of America; 2 Department of Medical Genetics, University of Antwerp, Antwerp, Belgium; 3 Department of Small Animal Medicine and Surgery, College of Veterinary Medicine, University of Georgia, Athens, Georgia, United States of America; 4 The Animal Neurology and Imaging Center, Algodones, New Mexico, United States of America; 5 Department of Veterinary Pathobiology, College of Veterinary Medicine and Biomedical Sciences, Texas A&M University, College Station, Texas, United States of America; 6 Department of Surgical and Radiological Sciences, School of Veterinary Medicine, University of California Davis, Davis, California, United States of America; 7 Department of Pathobiology, Faculty of Veterinary Medicine, Utrecht University, Utrecht, The Netherlands; 8 Section of Clinical & Comparative Neuropathology, Ludwig Maximilians University Munich, Munich, Germany; 9 Department of Small Animal Medicine, University of Leipzig, Leipzig, Germany; 10 Department of Biomedical Sciences, College of Veterinary Medicine, Cornell University, Ithaca, New York, United States of America; 11 Department of Clinical Sciences, College of Veterinary Medicine, North Carolina State University, Raleigh, North Carolina, United States of America; 12 Department of Veterinary Pathobiology, Veterinary Medical Diagnostic Laboratory, University of Missouri, Columbia, Missouri, United States of America; University of Sydney, Australia

## Abstract

Necrotizing meningoencephalitis (NME) affects toy and small breed dogs causing progressive, often fatal, inflammation and necrosis in the brain. Genetic risk loci for NME previously were identified in pug dogs, particularly associated with the dog leukocyte antigen (DLA) class II complex on chromosome 12, but have not been investigated in other susceptible breeds. We sought to evaluate Maltese and Chihuahua dogs, in addition to pug dogs, to identify novel or shared genetic risk factors for NME development. Genome-wide association testing of single nucleotide polymorphisms (SNPs) in Maltese dogs with NME identified 2 regions of genome-wide significance on chromosomes 4 (chr4:74522353T>A, p = 8.1×10^−7^) and 15 (chr15:53338796A>G, p = 1.5×10^−7^). Haplotype analysis and fine-mapping suggests that *ILR7* and *FBXW7*, respectively, both important for regulation of immune system function, could be the underlying associated genes. Further evaluation of these regions and the previously identified DLA II locus across all three breeds, revealed an enrichment of nominal significant SNPs associated with chromosome 15 in pug dogs and DLA II in Maltese and Chihuahua dogs. Meta-analysis confirmed effect sizes the same direction in all three breeds for both the chromosome 15 and DLA II loci (p = 8.6×10^–11^ and p = 2.5×10^−7^, respectively). This suggests a shared genetic background exists between all breeds and confers susceptibility to NME, but effect sizes might be different among breeds. In conclusion, we identified the first genetic risk factors for NME development in the Maltese, chromosome 4 and chromosome 15, and provide evidence for a shared genetic risk between breeds associated with chromosome 15 and DLA II. Last, DLA II and *IL7R* both have been implicated in human inflammatory diseases of the central nervous system such as multiple sclerosis, suggesting that similar pharmacotherapeutic targets across species should be investigated.

## Introduction

Necrotizing meningoencephalitis (NME) is a disease of young to middle aged dogs that results in a unique pattern of mononuclear inflammation and necrosis in the brain [1]. Initially identified in pug dogs in the late 1960 s [1], NME now has been described in several toy and small breeds: the Brussels Griffon [2], Chihuahua [3], Coton de Tulear [2], Maltese [4], Papillion [2], Pekingese [5], and shih tzu [2]. The clinical course of NME is rapid and progressive with presenting signs including seizures, altered mentation, behavior change, circling, and visual deficits. Presumptive diagnosis of NME can be made based on clinical presentation, magnetic resonance imaging, and cerebrospinal fluid analysis, but histopathological evaluation is needed for definitive diagnosis [6]. Unfortunately, NME often is fatal despite aggressive immunosuppressive treatment [7]. Although NME has been recognized for over 40 years, the etiopathogenesis is poorly understood. Initially, an infectious etiology was suspected [1] but thorough molecular investigations have ruled out infectious causes [8]–[10] and NME now is accepted to be an immune-mediated disease that occurs as a result of genetic susceptibility [11]; to date, no environmental triggers have been identified to contribute to disease onset [12]. Studies focused on elucidating the etiopathogenesis of NME are necessary to help improve diagnosis and treatment of this devastating disease.

Genetic susceptibility to NME has been presumed based on over-representation of the disease in certain breeds [1], [3], [4]. A 2009 study confirmed heritability of NME in pug dogs [13]. This was followed by a genome-wide scan of pug dogs with NME that showed a strong association with the dog leukocyte antigen (DLA) or major histocompatibility complex (MHC) class II region on chromosome 12 [14]. We then verified these findings with an independent cohort of pug dogs and identified a second region of association on chromosome 8 that contains the *STYX* and *GNPNAT1* genes [15]. The finding of MHC II association supports the accepted theory that NME is the result of an aberrant immune response [7] as numerous central nervous system (CNS) and non-CNS immune-mediated diseases have been associated with MHC II variants in dogs and people [11], [16]. Although the exact mechanisms by which MHC II variants contribute to disease are not understood, MHC II molecules themselves are known to determine the reactivity of T-cells towards foreign versus self epitopes [16]. Additionally, many genes within the MHC II complex, such as *TAP1* and *TAP2*, are important for normal immune function and antigen presentation [17].

Similar to NME, multiple sclerosis is an inflammatory disease of the CNS that has a guarded prognosis [18]. The discovery of an MHC II association in dogs with NME led to speculation that NME and multiple sclerosis may share a similar pathogenesis [14]. Although the clinical course of NME is more severe than classic forms of multiple sclerosis, NME shares clinical and pathological features with atypical forms of multiple sclerosis such as Marburg variant [14], [18]. However, it is important to note that demyelination, a key feature of multiple sclerosis pathology, is not present in NME [1], [18]. Despite this key difference, NME represents a spontaneous model of non-infectious CNS inflammation that may prove useful in investigations of multiple sclerosis.

The use of canine models to map disease loci has proven to be highly effective, with fewer markers and samples needed than in human populations. This is because each dog breed is a genetic isolate only a few hundred years old with long-range haplotypes across its entire genome [19]. We hypothesized that, similar to the pug breed, Maltese and Chihuahua dogs have genetic risk factors for NME development and that genetic risk for NME would be shared among breeds. To identify novel and shared genetic variants, we analyzed single nucleotide polymorphisms (SNPs) across the genome in Maltese and Chihuahua dogs and compared our results to an expanded population of the pug dogs that we previously interrogated [15].

## Results

### Association testing

**Figure 1:**
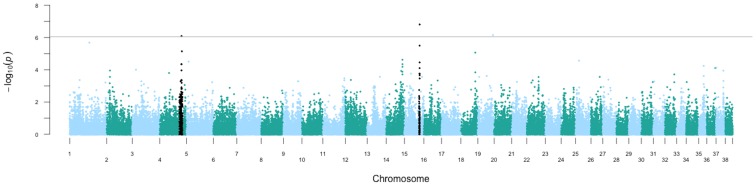
Manhattan plot of genome wide association analysis in Maltese dogs with necrotizing meningoencephalitis. The raw –log_10_ p-values for each SNP as determined by Fisher’s exact tests are plotted (y axis) against the chromosome position (x axis). The horizontal gray line represents the threshold for significant association after Bonferroni correction. Regions that reach genome wide significance are indicated in black.

**Figure 2:**
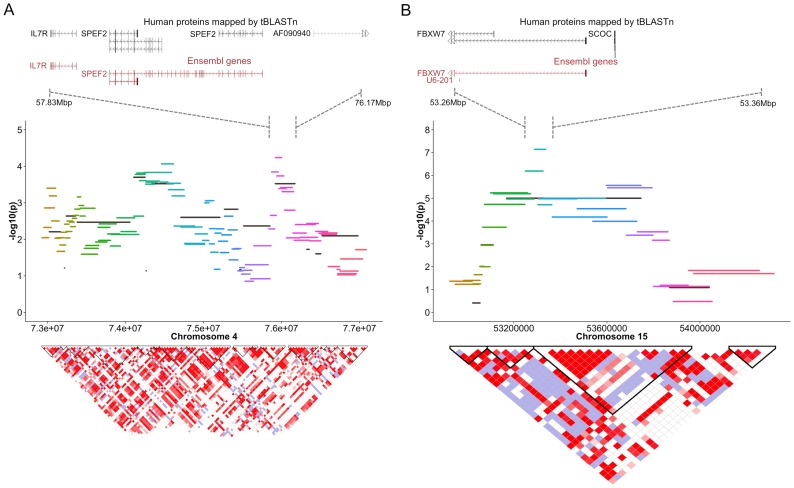
Fine mapping of genome wide significant regions on (A) chromosomes 4 and (B) 15 in Maltese dogs by haplotype analysis. The raw –log_10_ p-values for each haplotype test are plotted (y axis) against the chromosome position (x axis). The colored lines represent haplotypes formed by a 5-SNP sliding window across the region, and the black lines represent haplotypes formed by the underlying LD structure calculated by the 4-gamete rule. In the bottom, the LD between the SNPs in the region is shown (dark red = high D’; blue = low D’). The haploblocks based on the 4-gamete rule are indicated in black.

Association testing in Maltese dogs revealed two regions that reached genome wide significance on chromosome 4 (chr4∶74,522,353T>A, p = 8.07×10^−7^) and chromosome 15 (chr15∶53,338,796A>G, p = 1.55×10^−7^) (Table 1, Figure 1, File S2), while association testing in Chihuahua dogs did not reveal any regions of genome wide significance (Figure S1 in file S1). The region identified on chromosome 4 spreads about 10 Mega-basepairs (Mb), with the region of highest significance spanning approximately 4.4 Mb between chr4∶71,506,894-75,929,427 (Figure S2 in file S1). A tBLASTn query identified 44 human proteins mapped to this region of the canine genome (Table S1 in file S1, Figure 2). The second region on chromosome 15 spreads about 1.9 Mb between chr15∶51,567,064-53,471,253 and using tBLASTn, 19 human proteins mapped to this region of the canine genome (Table S1 in file S1, Figure 2). Additionally, all genes in these regions were prioritized based on ENDEAVOUR gene prioritization with a training set of human multiple sclerosis genes [20], which resulted in prioritization of *IL7R* on chromosome 4 and *FBXW7* on chromosome 15 (Table S1 in file S1, Figure 2).

**Table 1:** Most significantly associated SNPs from genome wide association analysis of Maltese dogs with necrotizing meningoencephalitis.

Chr	Position	SNP	P_raw_	BONF	FDR_BH	Max(T)	AF cases	AF controls	OR	L95	U95
4	74332455	BICF2G630168115	4.52E-05	1.000	0.157	0.696	0.82	0.31	9.818	2.972	32.440
4	74457236	BICF2G630168169	4.52E-05	1.000	0.157	0.696	0.82	0.31	9.818	2.972	32.440
**4**	**74522353**	**BICF2G630168244**	**8.07E-07**	**0.039**	**0.013**	**0.027**	**0.91**	**0.31**	**21.820**	**4.684**	**101.600**
4	75929427	BICF2P1021800	7.16E-06	0.348	0.058	0.199	0.00	0.49	0		
15	53260054	BICF2P1314960	3.52E-05	1.000	0.156	0.593	0.45	0.04	18.270	4.269	78.200
15	53272289	BICF2P956368	3.19E-06	0.155	0.031	0.097	0.50	0.04	22.330	5.361	93.040
**15**	**53338796**	**rs8954494**	**1.55E-07**	**0.008**	**0.008**	**0.005**	**0.60**	**0.04**	**33.500**	**7.763**	**144.600**

Chr: chromosome; P_raw_: Fisher exact test p-value; BONF: Bonferroni correction for multiple testing; FDR BH: false discovery rate of multiple testing; Max(T): Max(T) permutation of 10,000 permutations as corrected empirical p (EMP2) value; AF: allele frequency; OR: odds ratio; L95 and U95: upper and lower 95% confidence intervals for the OR. Coordinates are based on CanFam2.0 alignment. The SNPs with greatest significance are represented with bold lettering.

We next hypothesized that these regions of genome-wide significance identified in the Maltese breed also may contribute to disease development in the Chihuahua and pug breeds, which were also genotyped using the CanineHD beadchip (Illimina Inc, San Diego, USA). For chromosome 4, genotyping of 139 SNPs in Chihuahua dogs and 237 SNPs pug dogs only found 5 SNPs in each (3.5% and 1.5%, respectively) that reached nominal significance (p<0.05). However, for chromosome 15, genotyping of 55 SNPs in Chihuahua dogs and 75 SNPs in pug dogs found 3 (3.5%) and 18 (24%) that reached nominal significance, respectively. In the pug breed, this was much higher than the expected 5%, which suggests that this area of chromosome 15 also may be important for NME susceptibility in the pug breed.

**Figure 3:**
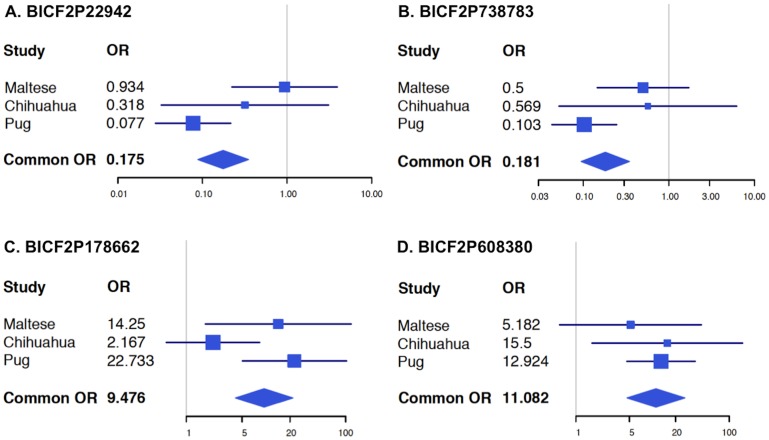
Forest plots of four SNPs in the dog leukocyte antigen II region on chromosome 12 across three toy breeds with necrotizing meningoencephalitis. (a) BICF2P22942: p = 1.57×10^−7^, OR = 0.18 (0.09–0.35), (b) BICF2P738783: p = 4.11×10^−8^, OR = 0.18 (0.10–0.34), (c) BICF2P178662: p = 1.11×10^−9^, OR = 9.48 (4.19–21.40), and (d) BICF2P608380: p = 8.6×10^−11^, OR = 11.08 (4.72–26.01). The 95% confidence interval for each study is represented by a horizontal line, and the point estimate is given by a square, the height of which is inversely proportional to the standard error of the estimate after each study. The summary OR is indicated by a diamond with horizontal limits as the confidence limits and height inversely proportional to its standard error. The meta-analyses for all these SNPs were significant and effect sizes were in the same direction.

Additionally, we hypothesized that the genetic risk loci previously identified in pug dogs on chromosome 12 and chromosome 8 [15] may contribute to disease susceptibility in the Maltese and Chihuahua breeds. In the Maltese, genotyping of 122 SNPs within the DLA II locus (chr12∶4,713,392-8,834,652) found 17 (13.9%) that were significant (p<0.05) (Figure S4 in file S1), which is higher than the expected 5%. The most significant SNP was BICF2P178662 chr12∶5,166,878 (p = 0.001) (Figure 3). In the Chihuahua, 120 SNPs were genotyped in the same region, of which 8 were significant (6.6%) (Figure S5 in file S1). The most significant SNP was BICF2P608380 chr12∶6,289,014 (p = 0.006) (Figure 3). On chromosome 8, where *STYX* and *GNPNAT1* are located (chr8∶31,736,206-32,225,068), 13 SNPs were analyzed in Maltese dogs and 9 SNPs were analyzed in Chihuahua dogs, but none reached significance.

### Fine-mapping and haplotype analysis

Fine-mapping of the regions on chr4 and chr15 identified in the Maltese was done by a haplotype analysis, using a sliding window of 5-SNPs across the region (Omnibus test; Figure S3 in file S1, File S2). Based on these data, two smaller regions were selected further for an in-depth haplotype analysis (Figure S3 in file S1; chr4∶73,000,000-77,000,000; chr15∶53,000,000-54,000,000). We performed haplotype-specific tests within the 5-SNP sliding window in these areas, and defined additional haplotypes based on the linkage disequilibrium (LD) structure in the region (Figure 2). This identified specific areas of haplotypes that are most significantly associated with NME. The most significant haplotype on chr4 is WIN66387 (BICF2P781476|BICF2P1021800|BICF2G630168994|BICF2G630169020|BICF2G630169045; CCGGG) and WIN66388 (BICF2P1021800|BICF2G630168994|BICF2G630169020|BICF2G630169045|BICF2P633927; CGGGA) (p = 5.78×10^−5^), located right adjacent on chr4∶75916205-76003694. These two haplotypes contain the *FLJ23577* or *SPEF2* gene (Homo sapiens sperm flagellar 2), which is important in the development of sperm, and specifically localized in germ and sertoli cells. However, this haplotype is also located only 55kbp upstream of *IL7R*, suggesting that this gene is the main factor in this region associated with NME in the Maltese. WIN66387, CCGGG, and WIN66388, CGGGA, were both present in 47% of the controls, and none of the cases. This suggests that these haplotypes are protective against NME, and other haplotypes at this position are at risk for NME (CGAGG and GAGGA, respectively).

The most significant haplotype on chr15 is p = 7.23×10^−8^; chr15∶53287510-53338796; WIN85935 (BICF2S23238125|TIGRP2P203637_rs8672515|BICF2S22948103|BICF2S23527645|TIGRP2P203665_rs8954494; GACGG). This haplotype contains the first part of the *FBXW7* gene and a pseudogene of the *SCOC* gene, which is found in multiple places of the canine genome.

The WIN85935, GACGG haplotype is present in 47% of the cases and 95% of the controls. Both other WIN85935 haplotypes are at risk for developing NME (GACGA; 15% of the cases, 0 of the controls; and AGAAA; present in 38% of the cases and 4% of the controls).

### Meta-analysis

To look more closely at the previously identified regions of significance, we performed meta-analysis on SNPs within these regions in Maltese, Chihuahua, and pug populations. We found similar effect sizes for all 3 breeds for the DLA II region of chromosome 12 (Figure 3) and the chromosome 15 locus (Figure S6 in file S1). For the DLA II region, Figure 3 shows the SNPs that were most significant in the Maltese (BICF2P178662), Chihuahua (BICF2P608380), and pug (BICF2P738783 and BICF2P22942) breeds. Power calculations show that the Maltese and Chihuahua data-sets have 52% and 38% power, respectively to detect the previous effect present in the pug when using an allelic test (p<0.05) [14], [21]. Meta-analysis across the entire genome only designated the DLA II region as genome wide significant (p = 1.18×10^−8^), followed by the region on chromosome 15 (p = 2.23×10^−6^).

## Discussion

We evaluated SNPs across the genome in three toy breeds susceptible to NME (Maltese, Chihuahua, and pug dogs) to further characterize genetic risk for this devastating neurological disease. Genome wide association in Maltese dogs identified risk loci associated with NME on chromosome 4 and chromosome 15, both of which contain genes of interest that are critical for normal immune system function. Association testing in Chihuahua dogs did not identify risk loci of genome wide significance, likely due to the small sample size. Additional evaluation of the regions identified on chromosome 4 and chromosome 15, as well as the genetic risk loci previously identified in pug dogs with NME [15], demonstrated that the risk locus on chromosome 15 and the DLA II complex on chromosome 12 likely contribute to genetic susceptibility for NME across breeds.

The most notable gene of interest within the region of significance on chromosome 4 is *IL7R*, which encodes for the interleukin (IL) 7 receptor alpha chain and is located about 68kb from significant marker BICF2P1021800 (Table 1) and only 55kb from the most significant region fine-mapped by haplotypes (Figure 2). This region is located right upstream of *IL7R*. *IL7R* is a member of the type I cytokine receptor family; it is critical for proliferation and survival of T- and B-lymphocytes as well as regulation of immune system function. Alterations in *IL7R* have not been associated with disease in dogs, but polymorphisms in *IL7R* have been demonstrated to contribute to non-MHC genetic risk in multiple sclerosis [22], [23]. Additionally, altered expression of the genes encoding IL7Rα and its ligand, IL7, has been found in the cerebrospinal fluid of individuals with multiple sclerosis [23]. Although a causal variant associated with *IL7R* has not been identified, this finding in Maltese dogs supports the hypothesis that NME and multiple sclerosis may share similar pathways for disease development.

Fine-mapping of the region of chromosome 15 showed that *FBXW7* is located in the haplotype most significantly associated with disease (Figure 2). The alpha isoform of *FBXW7* is ubiquitously expressed and has been shown to be an important attenuator of inflammatory signaling, in part due to suppression of CCAAT enhancer binding protein-delta (C/EBPδ) [24]. Reduced expression of C/EBPδ in mice is associated with increased expression of IL10 by dendritic cells with subsequent enhancement of regulatory T-cells and less severe clinical disease when experimental autoimmune encephalitis is induced by vaccination with myelin oligodendrocyte glycoprotein [25]. Reduced expression of C/EBPδ also has been associated with decreased expression of pro-inflammatory genes in neural tissue [26]. Another gene of interest on chromosome 15 is *LRBA* as deleterious mutations in *LRBA* have been associated with a syndrome of immune deficiency and autoimmunity in people [27].

In addition to the identification of novel risk loci in the Maltese breed, we identified shared risk associated with chromosome 15 as well as the DLA II locus [14], [15] by identification of enrichment of nominal significantly associated SNPs and meta-analysis. Importantly, the DLA II locus reached genome wide significance across all breeds when genome wide meta-analysis was performed. Since the power of the Maltese and Chihuahua datasets are low at 52% and 38%, respectively, larger sample sizes may more clearly demonstrate a DLA II association with NME in the Maltese and Chihuahua breeds. The identification of shared risk for NME development in multiple toy breeds should allow for fine mapping across breeds to identify more specific variants that result in disease development.

In conclusion, we discovered two susceptibility loci in Maltese dogs with NME, on chromosome 4 (chr4∶75916205-76003694; upstream of *IL7R*) and chromosome 15 (chr15∶53287510-53338796; *FBXW7)*, and evidence of shared risk for NME development in the Maltese, Chihuahua, and pug breeds. Currently, a test for genetic susceptibility of NME in pug dogs is commercially available to help with breeding programs [11]. The information presented here may help with development of similar tests in the Maltese and Chihuahua breeds. Ongoing investigations also are aimed at identification of causal variants for NME development with the long-term goal of developing an antemortem diagnostic test as well as targeted therapies. Further studies of these regions in other canine populations should provide an accurate estimate of the effect size that would lead to the implementation of a diagnostic test. Finally, the finding that *IL7R*, in addition to DLA II, may be associated with NME development supports previous theories that NME and multiple sclerosis may have similar pathogenic mechanisms. In the future, we anticipate that the insights gained from dogs with NME may help with development of more personalized and effective treatments for both NME and multiple sclerosis.

## Materials and Methods

### Inclusion criteria and sample collection

Samples were collected from Maltese, Chihuahua, and pug dogs with NME. Where possible, cases of NME had histopathological confirmation of disease diagnosis, which involved review of hematoxylin and eosin stained sections of brain tissue by a veterinary neuropathologist (BP and KM) for accurate diagnosis according to previous descriptive reports [1], [3], [4]. Tissue collected from these cases included archived formalin fixed paraffin embedded (FFPE) tissue and fresh frozen brain tissue stored at −80 degrees Celsius. These tissues initially were collected from client owned animals for owner requested necropsy. Use of these samples in this investigation was approved by the Clinical Research Committee at the University of Georgia, which serves as the Veterinary Teaching Hospital’s internal review board for all clinical research involving client owned animals.

When brain tissue was not available for histopathological confirmation, a presumptive diagnosis of NME was made using the following inclusion and exclusion criteria. Included dogs had to meet all of the following criteria: purebred Maltese or Chihuahua with pedigree information provided by the owner; 6 months to 7 years of age at time of disease onset; clinical signs present for less than 2 weeks; neuroanatomic localization to the prosencephalon with or without concurrent localization to the cerebellum or brainstem; MRI findings consistent with previously described reports as outlined below; a mononuclear pleocytosis of greater than 5 white blood cells per microliter in an atlantooccipital or lumbar cerebrospinal fluid (CSF) sample with no identifiable infectious agents or lymphoblasts; and negative antibody titers to *Neospora caninum*, *Toxoplasma gondii*, and *Ehrlichia canis* in serum or CSF. On MRI, dogs were required to have ill defined multifocal to diffuse, asymmetric lesions affecting the gray and white matter of the cerebral hemispheres that had to be hyperintense on T2-weighted and fluid-attenuated inversion recovery images and iso- to hypointense on T1-weighted images. Concurrent T2 hyperintensities in the cerebellum and brainstem could be present as long as lesions in these locations comprised less than 80% of the total lesion burden on MRI. Variable MRI findings included mass effect such as shift of the falx cerebri, brain herniation, and mild to moderate contrast enhancement of the brain parenchyma or leptomeninges. Dogs with strong contrast enhancement or peripheral rim enhancement were excluded [28]. Dogs also were excluded if they had evidence of systemic illness such as organ dysfunction, neoplasia, or infection based on a complete blood count, serum biochemistry, urinalysis, and three view thoracic radiographs; evidence of spinal cord disease on neurological exam or MRI; or evidence of neuromuscular disease on neurological exam.

Control dogs were determined by a veterinarian to be healthy at the time of sample collection had no history of autoimmune or neurological disease. Control dogs of the Maltese and Chihuahua breeds were all greater than 60 months of age at the time of sample collection and were followed for an additional 24 months to ensure they remained disease free until 84 months of age, after which time development of NME is unlikely. Control dogs of the pug breed ranged in age from 5 to 204 months (mean 60 months) at the time of sample collection. All control pugs aside from 2 that were lost to follow-up were followed for 60 months after sample collection to verify that they did not develop neurological or autoimmune disease; 13 control pugs all over the age of 84 months had died of other disease processes at the time of writing this paper. All presumptive NME cases and healthy controls were included with their owner’s consent and samples (1ml of whole blood or cheek swab of saliva) were obtained in strict accordance with good animal practice. The study protocol was approved by the University of Georgia Institutional Animal Care and Use Committee (IACUC number A2010 09-165-Y2-A0) and the IACUC of TGen Drug Development (TD2; protocol number 14010).

### Study population

In total, 196 samples from purebred Maltese, Chihuahua, and pug dogs were used for analysis. Samples from Maltese dogs were used for genome wide association and meta-analysis and included 8 with histopathologically confirmed NME, 3 with presumptive NME, and 38 healthy controls. Maltese dogs with NME included 5 females and 6 males. Healthy controls of the Maltese breed included 20 females and 18 males. Samples from Chihuahua dogs were used for genome wide association and meta-analysis and included 14 with histopathologically confirmed NME, 2 with presumptive NME, and 9 healthy controls. Chihuahua dogs with NME included 9 females and 7 males. Healthy controls of the Chihuahua breed included 6 females and 3 males. Additionally, 34 pug dogs with histopathologically confirmed NME and 88 healthy control pug dogs were utilized for genotyping and meta-analysis. These pug dogs were an expansion of the dataset used by Barber et al. in 2011 [15] and included 22 females and 12 males with NME as well as 48 females and 40 males that served as healthy controls.

### DNA preparation and genotyping

Genomic DNA was extracted from whole blood, saliva, fresh frozen brain tissue, or FFPE tissue sections. Extraction from EDTA blood samples and fresh frozen brain tissue was performed with the DNeasy Blood and Tissue Kit (Qiagen, Valencia, CA, USA). Saliva samples were extracted using the Oragene saliva extraction protocol (DNA Genotek, Ontario, Canada). Extraction from FFPE tissues utilized the QIAamp DNA FFPE Tissue Kit (Qiagen, Valencia, CA, USA) followed by whole genome amplification with the Ovation WGA FFPE system (NuGEN, San Carlos, USA). Samples were genotyped using the CanineHD beadchip, following manufacturer’s instructions (Illumina Inc, San Diego, USA).

### Statistical Analysis

A total of 173,662 SNPs were genotyped in all cases and controls and analysis was performed with PLINK v1.07 [29]. Pairwise identity by decent estimation was done to remove possibly related dogs (Pi_hat score >0.5 were excluded), leading to the exclusion of 1 Maltese control (Pi_hat score 0.64). Next, multidimensional scaling (MDS) with a minor allele frequency (MAF) of >0.10 and genotype frequency >97% was performed to determine population stratification, and 2 Maltese controls that were not clustered with the main population of dogs were excluded resulting in a final population of 11 Maltese NME cases and 35 Maltese controls for analysis. Further testing of population stratification was done on this sample set by plotting a QQ plot of observed against expected p-values, resulting in a genomic inflation factor of 1.025, or 1.0004 when corrected for the two associated regions (Figure S7 in file S1), demonstrating no significant population stratification in the sample set. Two affected pug dogs and 44 control pug dogs were excluded based on MDS, resulting in 32 NME cases and 44 controls for final analysis. No Chihuahuas were excluded based on population structure analysis, resulting in 16 NME cases and 9 controls for final analysis.

All had call rates of >80% and were included in the analysis. After excluding SNPs with a call rate <97%, MAF of <0.10, and Hardy-Weinberg equilibrium of >0.001 as well as SNPs on the X and Y chromosomes, a total of 57,222 SNPs were analyzed using Fisher’s exact tests for comparison of allelic frequency (for the Maltese dataset). A Bonferroni correction for multiple testing was performed with a resulting p-value of 8.7×10^−7^ across 57,222 SNPs for genome-wide significance. To further evaluate genome-wide significance MaxT permutation testing of 10,000 permutations and a false discover rate correction were applied [30]. All plots were created using R2.15.3 packages qqman and ggplot2 (http://www.r-project.org). Regions of interest were defined by SNPs reaching genome wide significance and surrounding SNPs with a suggestive p = 1×10^−4^ significance.

### Haplotype analysis

The regions on chromosome 4 and 15 in the Maltese were further fine-mapped by haplotype analysis. This was done with PLINK v1.07 [29], by using a 5-SNP sliding over the region of interest. In addition, haploblocks were chosen with Haploview based on the underlying LD structure of the region [31]. The four-gamete rule with a fourth haplotype frequency cutoff of 0.1 was used to define haploblocks based on LD [31].

### Meta-analysis

To calculate the common effect size of SNPs across the three breeds, meta-analysis and corresponding forest plots were performed using the R package Rmeta and R2.15.3 (http://www.r-project.org). A logistic regression model was used to estimate the common odds ratio with origin and genotype as independent variables and outcome as a dependent variable (the Mantel-Haenszel method). In addition, genome wide meta-analysis was performed using PLINK v1.07 [29]. Power calculations were done using the genetic power calculator [21], assuming 29% carrier frequency, a relative risk of 5.45 in the homozygotes, a disease frequency of 1%, and considering unscreened random controls [14].

## Supporting Information

File S1 **Supplementary Table and Figures.**
(DOCX)

File S2 **GWAS, meta-analysis and haplotype analysis results.** This file includes the GWAS results from the Maltese population, meta-analysis results and haplotype analysis data.
(ZIP)
