# Supplementary material for: Identification of Novel Genetic Risk Loci in Maltese Dogs with Necrotizing Meningoencephalitis and Evidence of a Shared Genetic Risk across Toy Dog Breeds
Source: PLoS One. 2014 Nov 13;9(11):e112755. doi: 10.1371/journal.pone.0112755 (PMC4231098; doi:10.1371/journal.pone.0112755)
Supplement: File S1 — Supplementary Table and Figures. (DOCX) [file pone.0112755.s001.docx]

**Supplementary Data**

**Table S1. Human proteins mapped to the canine genome (tBLASTn) at the regions of genome-wide significance on *Canis familiaris* (CFA) 4 and 15 in Maltese dogs with necrotizing meningoencephalitis.**

| **CFA4** | **CFA15** |
| --- | --- |
| AK097143 | AK097143 |
| AK127847 | AK127847 |
| AK128036 | AK131383 |
| BC036758 | ATP5J2 |
| C13orf12 | BC036758 |
| C6 | FBXW7 |
| C7 | LOC401622 |
| C9 | LRBA |
| CAPSL | NBPF11 |
| CARD6 | NBPF8 |
| DAB2 | NHRC2 |
| FLJ13231 | PET112L |
| FLJ23577 | RPL34 |
| FLJ25422 | RPL39 |
| FLJ30596 | RPS15A |
| FLJ39155 | RPS24 |
| FLJ40243 | RPS3A |
| FLJ40453 | SCOC |
| FYB | SH3D19 |
| GDNF |  |
| IL7R |  |
| JOSD3 |  |
| KRT18 |  |
| LIFR |  |
| LMBRD2 |  |
| LOC402176 |  |
| MGC70863 |  |
| NIPBL |  |
| NUP155 |  |
| OSMR |  |
| OSRF |  |
| PRKAA1 |  |
| PTGER4 |  |
| RICTOR |  |
| RPL10A |  |
| RPL21 |  |
| RPL23A |  |
| RPL37 |  |
| SKP2 |  |
| SLC1A3 |  |
| UBA52 |  |
| UGT3A1 |  |
| UGT3A2 |  |
| WDR70 |  |

**Figure S1:** **Manhattan plot of genome wide association analysis in Chihuahua dogs with necrotizing meningoencephalitis.** The raw –log10 p-values for each SNP as determined by Fisher’s exact tests are plotted (y axis) against the chromosome position (x axis). The horizontal gray line represents the threshold for significant association after Bonferroni correction.

**
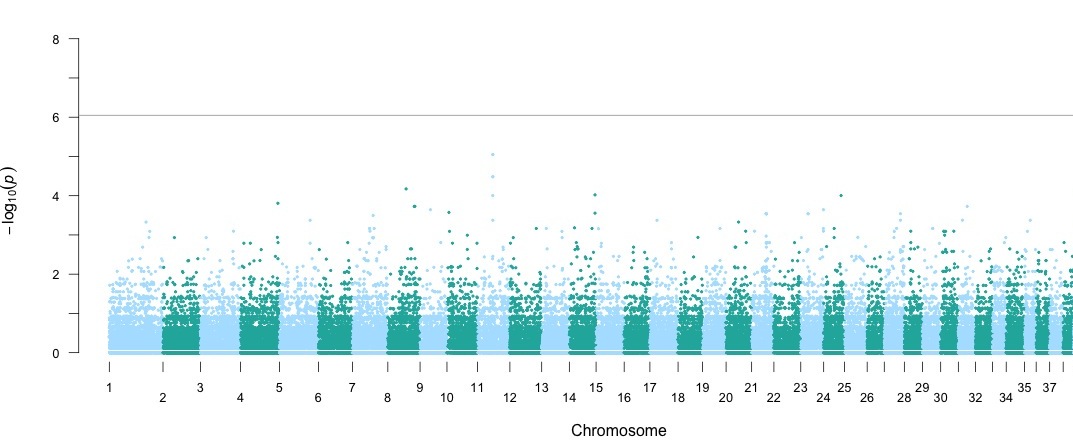
**

**Figure S2.** **Regional Manhattan plots and associated genes for the regions of genome wide significance on (A) chromosomes 4 and (B) 15 in Maltese dogs with necrotizing meningoencephalitis.** The raw –log_10_ p-values for each SNP as determined by Fisher’s exact tests are plotted (y axis) against the chromosome position (x axis). The associated regions are highlighted in blue. The horizontal gray line represents the threshold for significant association after Bonferroni correction.

**
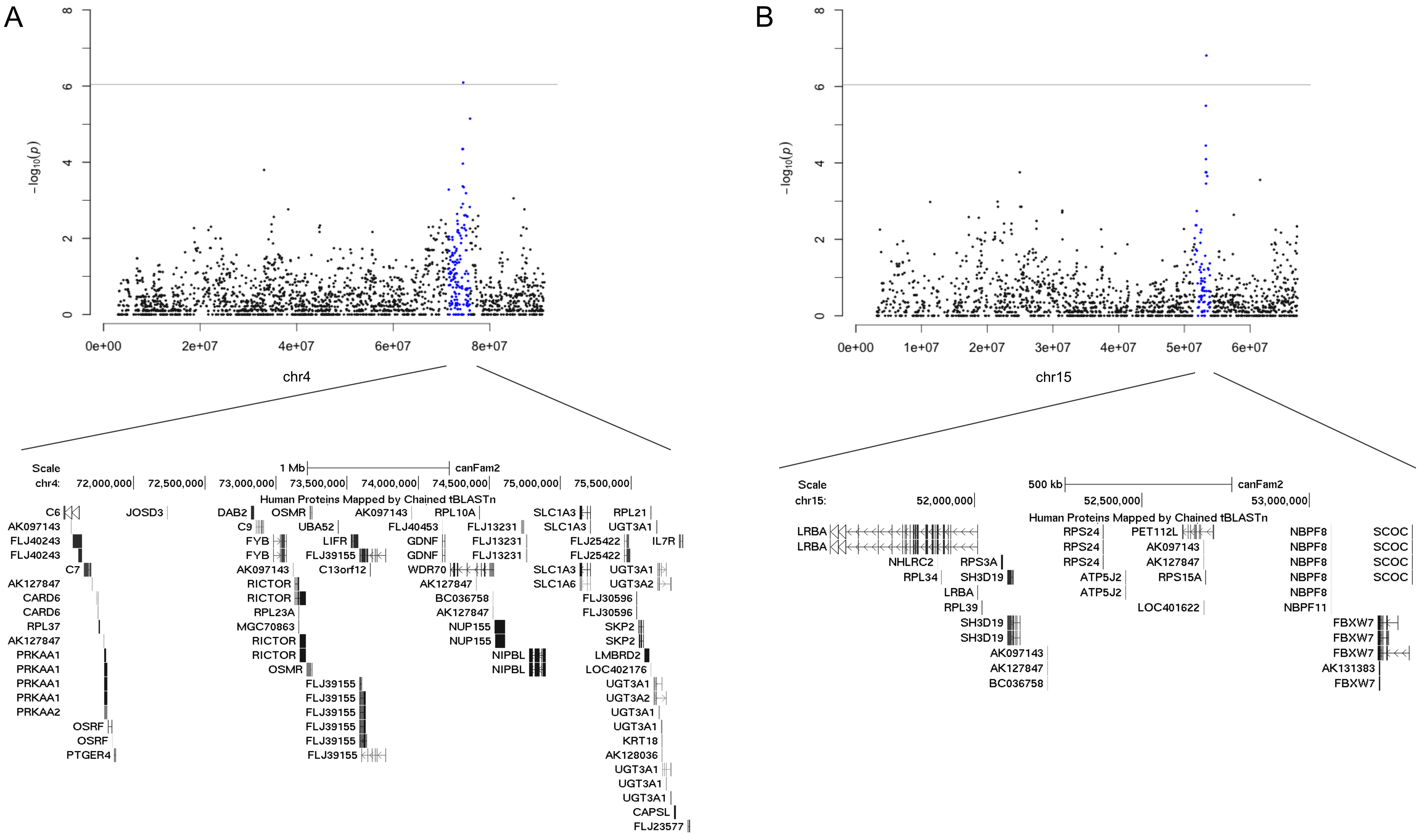
**

**Figure S3.** **Regional Haplotype analysis plots and associated genes for the regions of genome wide significance on (A) chromosomes 4 and (B) 15 in Maltese dogs with necrotizing meningoencephalitis.** The raw –log_10_ p-values for each SNP as determined by a 5-SNP sliding window association test are plotted (y axis) against the chromosome position (x axis). The regions highlighted were selected for further detailed haplotype analysis.

**
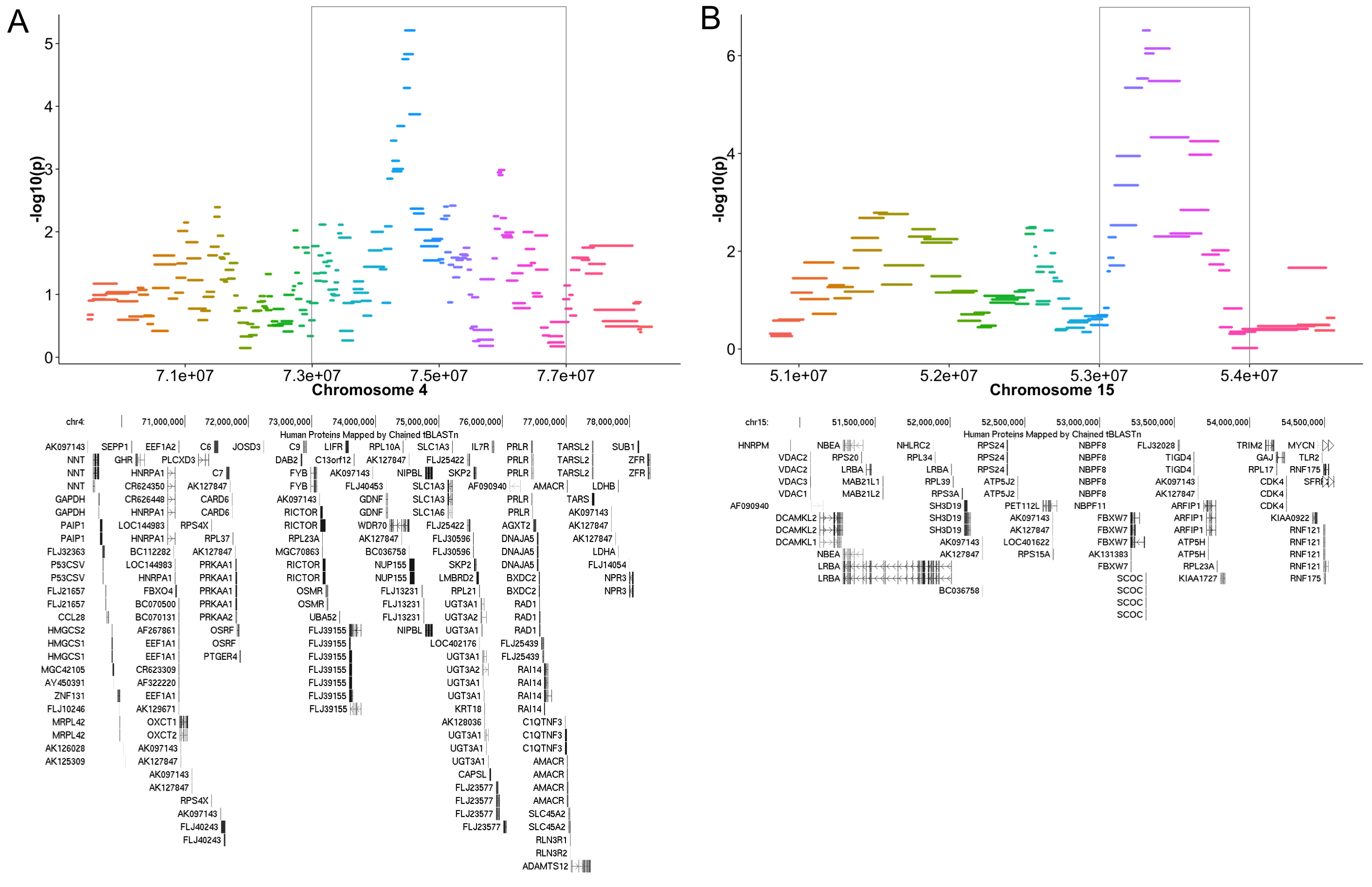
**

**Figure S4. Regional Manhattan plot for the dog leukocyte antigen II region on chromosome 12 in Maltese dogs with necrotizing meningoencephalitis.** The raw –log_10_ p-values for each SNP as determined by Fisher’s exact tests are plotted (y axis) against the chromosome position (x axis). The associated region is highlighted in blue. The horizontal gray line represents the threshold for significant association after Bonferroni correction. The blue line represents a p = 0.05 cut-off.


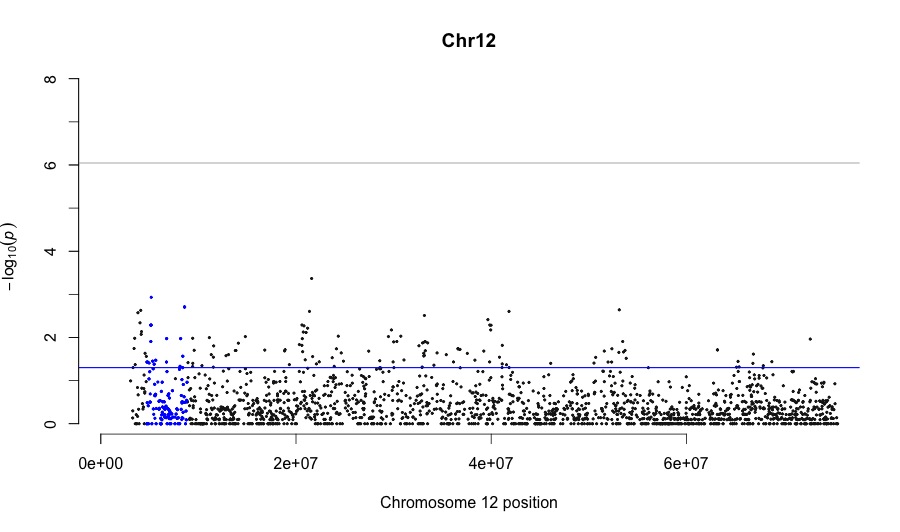


**Figure S5. Regional Manhattan plot for the dog leukocyte antigen class II region on chromosome 12 in Chihuahua dogs with necrotizing meningoencephalitis.** The raw –log_10_ p-values for each SNP as determined by Fisher’s exact tests are plotted (y axis) against the chromosome position (x axis). The associated region is highlighted in blue. The horizontal gray line represents the threshold for significant association after Bonferroni correction. The blue line represents a p = 0.05 cut-off.


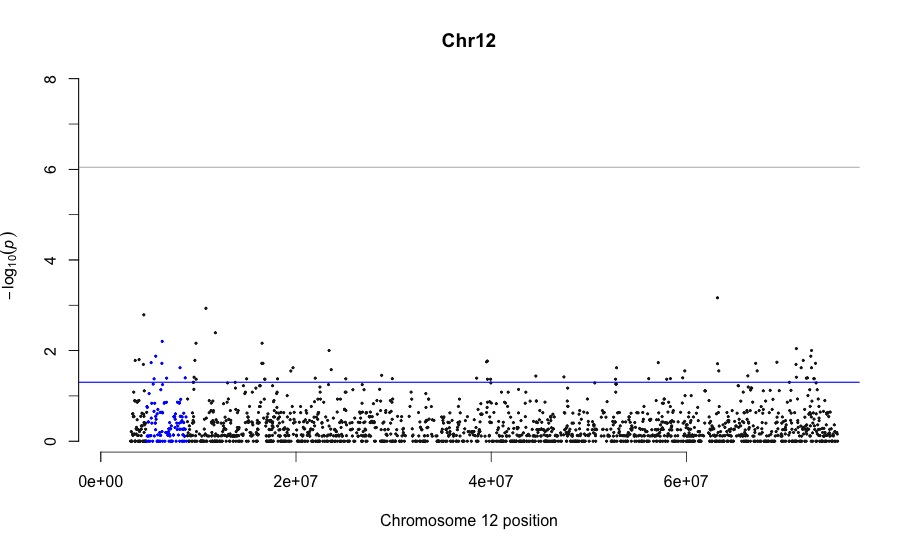


**Figure S6. Forest plots for the most significant SNPs on chromosome 4 (BICF2G630168244) and chromosome 15 (rs8954494) across three toy breeds with necrotizing meningoencephalitis.** (a) BICF2G630168244: p = 6.59 x 10^-4^, OR = 0.33 (0.17-0.65) and (b) TIGRP2P203665_rs8954494: p = 2.54 x 10^-7^, OR = 7.25 (2.94-17.91). The 95% confidence interval for each study is represented by a horizontal line and the point estimate is given by a square, the height of which is inversely proportional to the standard error of the estimate after each study. The summary odds ratio is indicated by a diamond, with horizontal limits as the confidence limits and height inversely proportional to its standard error. The meta-analyses for all these SNPs are significant and effect sizes are in the same direction for chromosome 15.


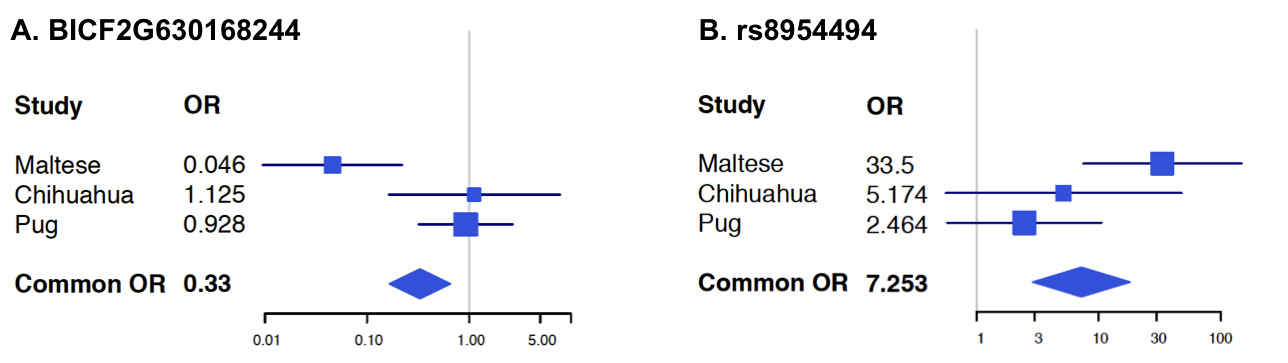


**Figure S7. QQ plot for Maltese dogs evaluated by genome wide association.** The plot compares expected versus observed –log_10_ p-values for all 173,662 SNPs included in genome wide association with the red line corresponding to the null hypothesis of no association.
